# Direct oral anticoagulants versus vitamin K antagonists after recent ischemic stroke in patients with atrial fibrillation

**DOI:** 10.1002/ana.25489

**Published:** 2019-04-30

**Authors:** David J. Seiffge, Maurizio Paciaroni, Duncan Wilson, Masatoshi Koga, Kosmas Macha, Manuel Cappellari, Sabine Schaedelin, Clare Shakeshaft, Masahito Takagi, Georgios Tsivgoulis, Bruno Bonetti, Bernd Kallmünzer, Shoji Arihiro, Andrea Alberti, Alexandros A. Polymeris, Gareth Ambler, Sohei Yoshimura, Michele Venti, Leo H. Bonati, Keith W. Muir, Hiroshi Yamagami, Sebastian Thilemann, Riccardo Altavilla, Nils Peters, Manabu Inoue, Tobias Bobinger, Giancarlo Agnelli, Martin M. Brown, Shoichiro Sato, Monica Acciarresi, Hans Rolf Jager, Paolo Bovi, Stefan Schwab, Philippe Lyrer, Valeria Caso, Kazunori Toyoda, David J. Werring, Stefan T. Engelter, Gian Marco De Marchis, on behalf of the CROMIS‐2, RAF, RAF‐DOAC, SAMURAI, NOACISP LONGTERM, Erlangen and Verona registry collaborators

**Affiliations:** ^1^ Neurology and Stroke Center University Hospital Basel and University of Basel Basel Switzerland; ^2^ Stroke Research Center, Department of Brain Repair and Rehabilitation UCL Institute of Neurology and The National Hospital for Neurology and Neurosurgery London United Kingdom; ^3^ Neurology and Stroke Center, Inselspital University Hospital Bern Bern Switzerland; ^4^ Stroke Unit and Division of Cardiovascular Medicine University of Perugia Perugia Italy; ^5^ Department of Cerebrovascular Medicine National Cerebral and Cardiovascular Center Suita Japan; ^6^ Department of Neurology University of Erlangen‐Nuremberg Erlangen Germany; ^7^ Stroke Unit–Department of Neuroscience Azienda Ospedaliera Universitaria Integrata Verona Italy; ^8^ Clinical Trial Unit, University Hospital Basel Basel Switzerland; ^9^ Second Department of Neurology, National & Kapodistrian University of Athens School of Medicine “Attikon” University Hospital Athens Greece; ^10^ Department of Neurology University of Tennessee Health Science Center Memphis TN; ^11^ Department of Statistical Science UCL London United Kingdom; ^12^ Institute of Neuroscience & Psychology, University of Glasgow Queen Elizabeth University Hospital Glasgow United Kingdom; ^13^ Neurorehabilitation Unit, University Center for Medicine of Aging and Rehabilitation Basel, Felix Platter Hospital University of Basel Basel Switzerland; ^14^ Neuroradiological Academic Unit, Department of Brain Repair & Rehabilitation, University College London Institute of Neurology London United Kingdom

## Abstract

**Objective:**

We compared outcomes after treatment with direct oral anticoagulants (DOACs) and vitamin K antagonists (VKAs) in patients with atrial fibrillation (AF) and a recent cerebral ischemia.

**Methods:**

We conducted an individual patient data analysis of seven prospective cohort studies. We included patients with AF and a recent cerebral ischemia (<3 months before starting oral anticoagulation) and a minimum follow‐up of 3 months. We analyzed the association between type of anticoagulation (DOAC versus VKA) with the composite primary endpoint (recurrent ischemic stroke [AIS], intracerebral hemorrhage [ICH], or mortality) using mixed‐effects Cox proportional hazards regression models; we calculated adjusted hazard ratios (HRs) with 95% confidence intervals (95% CIs).

**Results:**

We included 4,912 patients (median age, 78 years [interquartile range {IQR}, 71–84]; 2,331 [47.5%] women; median National Institute of Health Stroke Severity Scale at onset, 5 [IQR, 2–12]); 2,256 (45.9%) patients received VKAs and 2,656 (54.1%) DOACs. Median time from index event to starting oral anticoagulation was 5 days (IQR, 2–14) for VKAs and 5 days (IQR, 2–11) for DOACs (*p* = 0.53). There were 262 acute ischemic strokes (AISs; 4.4%/year), 71 intracranial hemorrrhages (ICHs; 1.2%/year), and 439 deaths (7.4%/year) during the total follow‐up of 5,970 patient‐years. Compared to VKAs, DOAC treatment was associated with reduced risks of the composite endpoint (HR, 0.82; 95% CI, 0.67–1.00; *p* = 0.05) and ICH (HR, 0.42; 95% CI, 0.24–0.71; *p* < 0.01); we found no differences for the risk of recurrent AIS (HR, 0.91; 95% CI, 0.70–1.19; *p* = 0.5) and mortality (HR, 0.83; 95% CI, 0.68–1.03; *p* = 0.09).

**Interpretation:**

DOAC treatment commenced early after recent cerebral ischemia related to AF was associated with reduced risk of poor clinical outcomes compared to VKA, mainly attributed to lower risks of ICH. **ANN NEUROL 2019;85:823–834.**

Oral anticoagulation is effective in the prevention of ischemic stroke and systemic embolism in patients with atrial fibrillation (AF).^1^, ^2^ Vitamin K antagonists (VKAs) inhibiting the production of several coagulation factors in the liver have been the only option for long‐term oral anticoagulation for many years.^1^ Direct oral anticoagulants (DOACs) including the thrombin inhibitor, dabigatran,^3^ and the factor Xa inhibitors,^4^ apixaban, edoxaban, and rivaroxaban, have been proven to be at least as effective in preventing ischemic stroke and systemic embolism in patients with AF while having a lower risk of symptomatic intracranial hemorrhage (ICH).^2^, ^5^, ^6^, ^7^, ^8^ Patients with ischemic stroke and AF are at high risk for early recurrent acute ischemic stroke (AIS),^9^, ^10^ which may be as high as 13% within the first 10 days in patients not treated with oral anticoagulants.^11^ Among patients in the control (no treatment) groups of the randomized International Stroke Trial, the rate of recurrent AIS within the first 14 days was still as high as 4.5% and 4.9%, respectively.^12^ Risk of ICH in this population, and the effect of early anticoagulation, is unclear^13^: To minimize the risk of ICH, all randomized controlled trials^5^, ^6^, ^7^, ^8^ (RCTs) comparing DOAC and VKA in patients with AF excluded patients with a recent ischemic stroke for arbitrary time periods ranging from 7 to 14 days for mild stroke, up to 3 to 6 months for severe strokes.^11^ Actually, in patients with a history of ischemic stroke enrolled in one of the RCTs,^14^, ^15^, ^16^, ^17^ the delay between the stroke and enrollment in the trial was rather long: In ROCKET‐AF^16^ (Rivaroxaban‐once daily, oral, direct factor Xa inhibition compared with vitamin K antagonism for prevention of stroke and Embolism Trial in Atrial Fibrillation), median delay was 551 days, and in ARISTOTLE^15^ (Apixaban for Reduction in Stroke and Other Thromboembolic Events in Atrial Fibrillation), only 33% of patients were enrolled within 1 year of stroke. In clinical practice, DOACs are often commenced earlier than in the aforementioned RCTs,^13^ yet little is known about safety and effectiveness of this approach.^18^, ^19^, ^20^, ^21^, ^22^, ^23^, ^24^, ^25^ Thus, we compared the clinical benefit of DOAC and VKA in patients having AF with a recent ischemic stroke or transient ischemic attack (TIA).

## Materials and Methods

### *Study Design*

As a joint collaborative initiative of seven European and Japanese prospective, observational cohort studies, we performed a pooled individual patient data analysis combining data of all consecutive patients in the participating studies. Three authors (D.J.S., S.T.E., and G.M.D.M.) searched PubMed and MEDLINE between January 1, 2012 and July 2017, with the terms “DOAC, VKA, atrial fibrillation and stroke or TIA.” We selected peer‐reviewed prospective, observational studies published in English based on real‐life cohorts in which DOAC or VKA were administered within 3 months after the index stroke or TIA. The principal investigators of these studies were contacted and invited to participate in a pooled individual patient data analysis. All invited centers agreed to participate. The following studies were included: the single center prospective cohort studies from Verona/Italy,^19^ Erlangen/Germany,^20^ Basel/Switzerland (“Novel oral anticoagulants in stroke patients”/NOACISP),^23^ and the multicenter cohort studies “Early Recurrence and Cerebral Bleeding in Patients With Acute Ischemic Stroke and Atrial Fibrillation” (RAF^21^ and RAF‐NOAC^22^; 29 centers in Europe and Asia), “The Clinical Relevance of Microbleeds in Stroke study” (CROMIS‐2; 79 centers in the UK and one in the Netherlands),^26^, ^27^ and “The Stroke Acute Management with Urgent Risk‐factor Assessment and Improvement‐Non‐Valvular Atrial Fibrillation Study” (SAMURAI‐NVAF; 18 centers in Japan).^18^, ^24^, ^28^ Details about the participating studies can be obtained from Table 1 (collaborators are listed in Supplementary Table 1). Study quality and risk of bias were critically appraised based on the scheme suggested by the Cochrane collaboration (“Tool to Assess Risk of Bias in Cohort Studies”; available at: http://methods.cochrane.org/sites/methods.cochrane.org.bias) and the Newcastle‐Ottawa Scale (available at: http://www.ohri.ca/programs/clinical_epidemiology/oxford.asp) by two authors (D.J.S. and G.M.D.M.). Disagreement between reviewers was resolved by collegial discussion. Our analysis was conducted with respect to the STROBE criteria for observational studies.^29^

**Table 1:** Single‐ and Multicenter Studies Participating in the Individual Patient Data Analysis

	Study Period	Patients Contributed to Final Cohort	Maximum Follow‐up Period^a^
Single center			
Verona (Italy)^19^	2013–2015	230	3‐month
Erlangen (Germany)^20^	2011–2013	337	Up to 1 year
NOACISP (Basel/Switzerland)^23^	2012–2017	518	Up to 3.8 years
Multicenter			
RAF (29 centers in Europe/Asia)^21^	2012–2014	572	3‐month
RAF‐NOAC (29 centers in Europe/Asia)^22^	2014–2016	963	3‐month
SAMURAI‐NVAF (18 centers in Japan)^24^	2011–2014	1,137	Up to 3.5 years
CROMIS‐2 (80 centers in the UK and one in the Netherlands)^26^	2011–2015	1,261	Up to 5.4 years

a Minimum follow‐up period of 3 months for all studies.

### *Inclusion and Exclusion Criteria*

We included patients with: (1) AIS (defined as a focal neurological deficit with acute onset and presence of a corresponding lesion on diffusion weighted [DWI] magnetic resonance imaging [MRI] or, if no MRI was acquired, signs of early ischemic injury on computed tomography [CT]) or TIA (defined as an acute‐onset focal neurological deficit of presumed ischemic origin without a corresponding lesion on DWI or, if no MRI was acquired, lasting less than 24 hours); (2) diagnosis of nonvalvular AF, either known before the index event or detected after the event; (3) oral anticoagulation with DOAC or VKA, either continued (for those already on anticoagulation on admission), started, or resumed within 3 months after the index event; (4) prospective follow‐up for at least 3 months or longer after the index event for the presence or absence of (i) recurrent AIS, (ii) ICH, and (iii) death (any cause). Patients who died before the first planned follow‐up (ie, within the first 3 months following the index event) were still included in the study.

We excluded patients with (1) mechanical heart valves; (2) rheumatic or severe mitral valve stenosis; and (3) oral anticoagulation started later than 3 months after the index event, or with missing information on oral anticoagulants initiation date.

### *Data Collection and Baseline Data*

Data were collected as done in previous published studies^30^, ^31^: Briefly, local investigators filled in standardized forms with predefined variables using individual patient data from their corresponding study database. Completed forms were collected at the coordinating center in Basel, where the pooled analysis was performed. The corresponding authors had full access to all the data in the study and take responsibility for its integrity and the data analysis.

### *Baseline Data*

The following baseline variables were recorded and provided by the participating studies: age, sex, and type of index event (AIS or TIA); antithrombotic treatment (no treatment, antiplatelet agents, VKA, or DOAC) before index event; type of anticoagulation after index event (VKA or DOAC), time from index event to first dose of VKA or DOAC; stroke severity on admission as assessed by the National Institutes of Health Stroke Scale (NIHSS)^32^; and use of intravenous thrombolysis or endovascular treatment for index stroke. DOAC therapy was defined as one of the following drugs and dosages: apixaban 2.5 or 5mg twice‐daily (bid); dabigatran 110 or 150mg bid; edoxaban 30 or 60mg once‐daily (od); or rivaroxaban 15 or 20mg od (10or 15mg od in Japan according to the results from a domestic trial^33^). VKA therapy was defined as treatment with phenprocoumon (NOACISP, Erlangen) or warfarin (SAMURAI‐NVAF, CROMIS‐2, RAF/RAF‐DOAC, and Verona). Choice of treatment was up to the decision of the treating physician.

The following risk factors were collected: history of ischemic stroke; history of ICH; diabetes mellitus; hypertension (defined as treated/controlled and untreated/uncontrolled hypertension); hypercholesterolemia; impaired renal function (defined as creatinine clearance of <60ml/min/1.73m^2^ using the Chronic Kidney Disease Epidemiology Collaboration [CKD‐EPI] equation^34^); current smoking; concomitant use of antiplatelet drugs or statins; and the CHA_2_DS_2‐_VASc^35^ and HAS‐BLED^36^ scores (designed to predict future AIS and major bleeding complications, respectively).

### *Follow‐up*

We included only studies with a planned follow‐up of at least 3 months after index event for (1) recurrent AIS defined as new neurological symptoms and evidence for ischemic stroke on CT or MRI, (2) ICH defined as new neurological symptoms associated with the detection of ICH on CT or MRI as defined within the International Society on Thrombosis and Haemostasis criteria,^37^ and (3) all‐cause mortality (including fatal AIS or ICH). International normalized ratio (INR) values at outcome event in patients on VKA was collected if available.

### *Outcome*

The primary outcome was the occurrence of the composite endpoint of recurrent AIS, ICH, and mortality. Secondary endpoints were the occurrence of each of these components separately.

### *Statistical Analysis*

We compared demographic and clinical baseline characteristics among patients in the two groups of anticoagulants using the Pearson χ2 test for categorical variables and the Mann–Whitney *U* test for continuous variables. An α‐level of 0.05 was used to determine statistical significance. Statistical analyses were carried out using R^38^ (R Foundation for Statistical Computing) and SPSS software (Version 25; IBM Corp, Armonk, NY). We calculated the annualized rate of outcome events (=total of observed events/patient‐years of follow‐up).

To assess the association between the type of anticoagulation (DOAC versus VKA) and the primary composite endpoint, time to endpoint, was modeled using a mixed‐effects Cox proportional hazards regression model to compute hazard ratios (HRs) with 95% confidence intervals (95% CIs). For competing risks of the secondary endpoints, the Fine‐Gray model was used.^39^ For the primary composite endpoint and for each secondary endpoint separately, we compared time until the first occurrence of an event. Only events occurring after starting VKA or DOAC were used. Type of anticoagulation (DOAC versus VKA) was included as a fixed effect. The analysis was adjusted for age, NIHSS at onset, history of stroke or TIA (ie, before the index event), history of ICH, sex, diabetes mellitus, smoking, treatment with intravenous thrombolysis for index event, and hypertension as fixed effects.” Cohort Study Identifier” (seven options) was included as a random effect. As a post‐hoc analysis, we further dichotomized the DOAC group into DOACs with od intake (edoxaban and rivaroxaban, DOAC_od_) and those with bid intake (apixaban and dabigatran, DOAC_bid_) and compared them separately with VKA.

We assessed treatment effects in the following predefined subgroups: (1) patients with minor stroke or TIA (defined as NIHSS ≤3)^40^; (2) patients with severe stroke (defined as NIHSS >15)^41^; (3) elderly patients (defined as aged ≥80 versus <80 years); (4) patients with impaired renal function (defined as creatinine clearance of <60ml/min/1.73m^2^ using the CKD‐EPI equation^34^); (5) patients treated with acute recanalization therapies for the index stroke (intravenous thrombolysis and/or endovascular therapy); and (6) patients started on anticoagulation earlier than in all phase‐3 RCTs regarding the DOACs (≤7 versus >7 days after index event).^23^ Thereby, each binary variable indicating a subgroup was included in a separate model and the interaction term between the covariate and the treatment was estimated. Furthermore, the same covariates as described for the main analysis were used as additional covariates. However, in the models for the subgroups “minor stroke” and “severe stroke,” NIHSS at onset was not used as a continuous covariate because dichotomized NIHSS was already used to define the subgroups. Similarly, the covariate age was replaced for the subgroup of elderly patients when estimating the interaction term. A significant interaction term indicates that the estimated difference between treatments differs between the subgroups. The estimated HRs are presented as forest plots. Missing values were not imputed, and analyses were performed using only patients without missing values in the relevant variables. However, as a sensitivity analysis, we repeated the analysis using multiple imputation for missing values^42^ using multivariate imputation by chained equations. Thereby, all available data were used as predictors for each target variable (sometimes called massive imputation). In total, five imputed data sets were constructed and analysed. The results were pooled according to Rubin's rule, using the Barnard‐Rubin adjusted degrees of freedom for small samples. In addition, we performed a propensity‐score‐weighted (propensity score matching; PSM) analysis using the R‐package “twang.” Thereby, boosted regression was used to estimate the propensity scores, including the same variables as described for the main analysis. The mean Kolmogorov‐Smirnov statistic was used as balance criteria used to tune the propensity score model. The composite endpoint as well as each of its components (recurrent AIS, ICH, and mortality) were presented in Kaplan‐Meier curves and cumulative incidence functions for competing risks.

### *Ethics*

The NOACISP LONG‐TERM registry and the current analysis of pooled individual patient data were approved by the ethics committee in Basel, Switzerland (EKNZ 2014‐027). Patients provided written consent for participation in NOACISP LONG‐TERM. The requirement for additional local ethical approval differed among participating centers and was acquired by the local principal investigator as well as written informed consent by the patient, if necessary. CROMIS‐2 was approved by the National Research Ethics Committee, London Queen Square. Patients with capacity gave informed written consent. When patients could not consent, we obtained written consent from a proxy as defined by relevant local legislation. The SAMURAI‐NVAF registry and the collaboration with the joint initiative were approved by the ethics committee in the National Cerebral and Cardiovascular Center (M23‐18‐3 and M29‐077).

## Results

**Figure 1:**
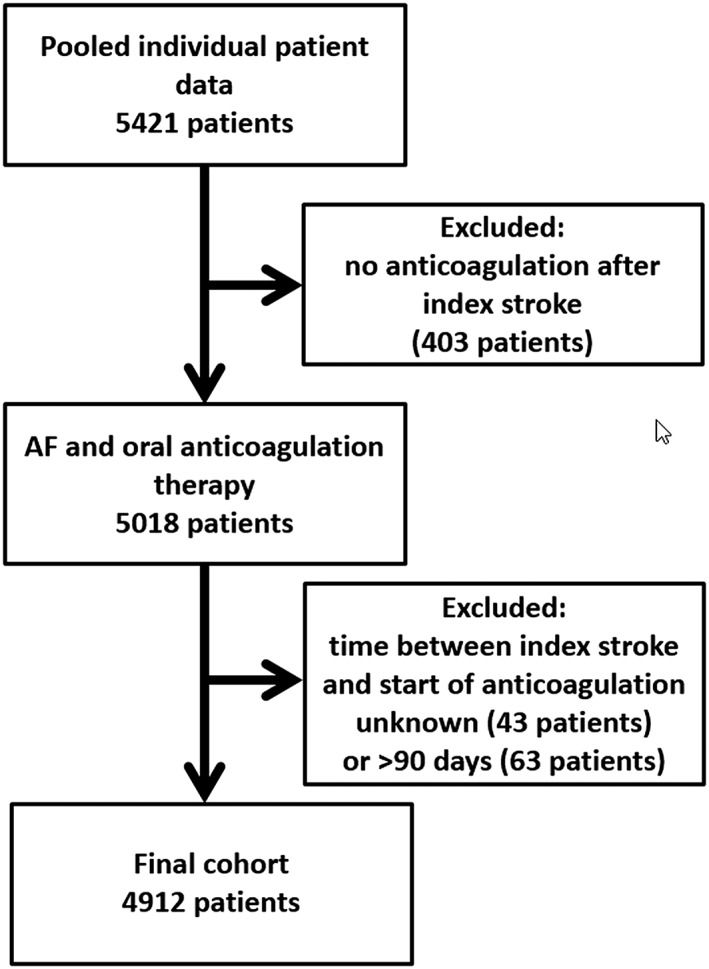
Study flow chart.

The final cohort comprised of 4,912 patients (see study flow chart Fig 1). Median age was 78 years (interquartile range [IQR], 71–84) and 2,331 (47.5%) patients were female. The index event was ischemic stroke in 4,739 patients (96.5%). Before the index event, 2,658 (54.1%) patients had no antithrombotic treatment, 429 (8.7%) were on antiplatelet agents, 607 (12.4%) on VKA, and 1,153 (23.5%) on any DOAC. After the index event, 2,256 (45.9%) patients received VKA and 2,656 (54.1%) received any DOAC. In the whole cohort, median time from index stroke to start of oral anticoagulation was 5 days (IQR, 2–12). There was no difference in median time from index stroke to starting oral anticoagulation between patients receiving VKA (median, 5 days; IQR, 2–14) and DOAC (median, 5 days; IQR, 2–11; *p* = 0.53). Overall, 3,993 patients (81.3%) were started on VKA or DOAC within the first 14 days after the index stroke. Overall, risk of bias was medium to low (Supplementry Table 2). Only one study^19^ (contributing 230 patients = 4.7% of the study population) was at high risk of bias because it did not include any patients on VKA (no controls).

### *Baseline Data and Demographics*

Baseline data of the DOAC and VKA cohorts are displayed in Table 2. To summarize, patients receiving DOACs had a lower NIHSS at baseline, a lower prevalence of diabetes mellitus, and more often received intravenous thrombolysis for the index stroke. Patients treated with VKA were more often current smokers.

**Table 2:** Baseline Characteristics of Patients With DOAC and VKA Therapy

	VKA n = 2,256	DOAC n = 2,656	*p*
Demographics			
Age in years	78 (71–84)	77 (71–84)	0.17
Sex female	1,060 (47.0%)	1,274 (48.0%)	0.35
Index stroke			
Acute ischemic stroke	2,178 (96.5%)	2,561 (96.4%)	0.82
TIA	78 (3.5%)	95 (3.6%)	
NIHSS at onset	6 (2–16)	4 (2–10)	<0.001^**^
Intravenous thrombolysis	385 of 2,192 (17.6%)	626 of 2,630 (23.8%)	<0.001^**^
Endovascular treatment	89 of 2,256 (3.9%)	90 of 2,656 (3.4%)	0.30
Time from index stroke to start of anticoagulation therapy (in days)	5 (2–14)	5 (2–11)	0.53
Risk factors			
History of ischemic stroke (before index event)	544 of 2,255 (24.1%)	600 of 2,655 (22.6%)	0.21
History of intracranial hemorrhage	26 of 1,701 (1.5%)	19 of 1,744 (1.1%)	0.26
Diabetes mellitus	610 of 2,251 (27.1%)	596 of 2,651 (22.5%)	<0.001^**^
Hypertension	1,669 of 2,242 (74.4%)	2,002 of 2,648 (75.6%)	0.35
Hypercholesterolemia	842 of 2,208 (38.1%)	739 of 1,826 (40.5%)	0.13
Impaired renal function^a^	529 of 1,717 (30.8%)	530 of 1,748 (30.3%)	0.78
Current smoking	325 of 2,213 (14.7%)	456 of 2,571 (17.7%)	0.04^*^
CHA_2_DS_2‐_Vasc	5 (4–6)	5 (4–6)	0.43
HAS‐BLED	3 (3–4)	3 (2–4)	0.21
Concomitant antiplatelet agents	826 of 2,073 (39.8%)	826 of 2,207 (37.4%)	0.10
Concomitant statins	230 of 935 (24.6%)	280 of 1,036 (27.0%)	0.22

Categorical variables are given in number of patients having the characteristic/total patients available for analysis and (%).

Continuous variables are displayed in median (interquartile range).

a Impaired renal function defined as creatinine clearance of <60ml/min/1.73m^2^.

^*^ *p* < 0.05; ^**^ *p* < 0.001.

TIA = transient ischemic attack; NIHSS = National Institute of Health Stroke Severity Scale.

### *Primary and Secondary Endpoints*

**Figure 2:**
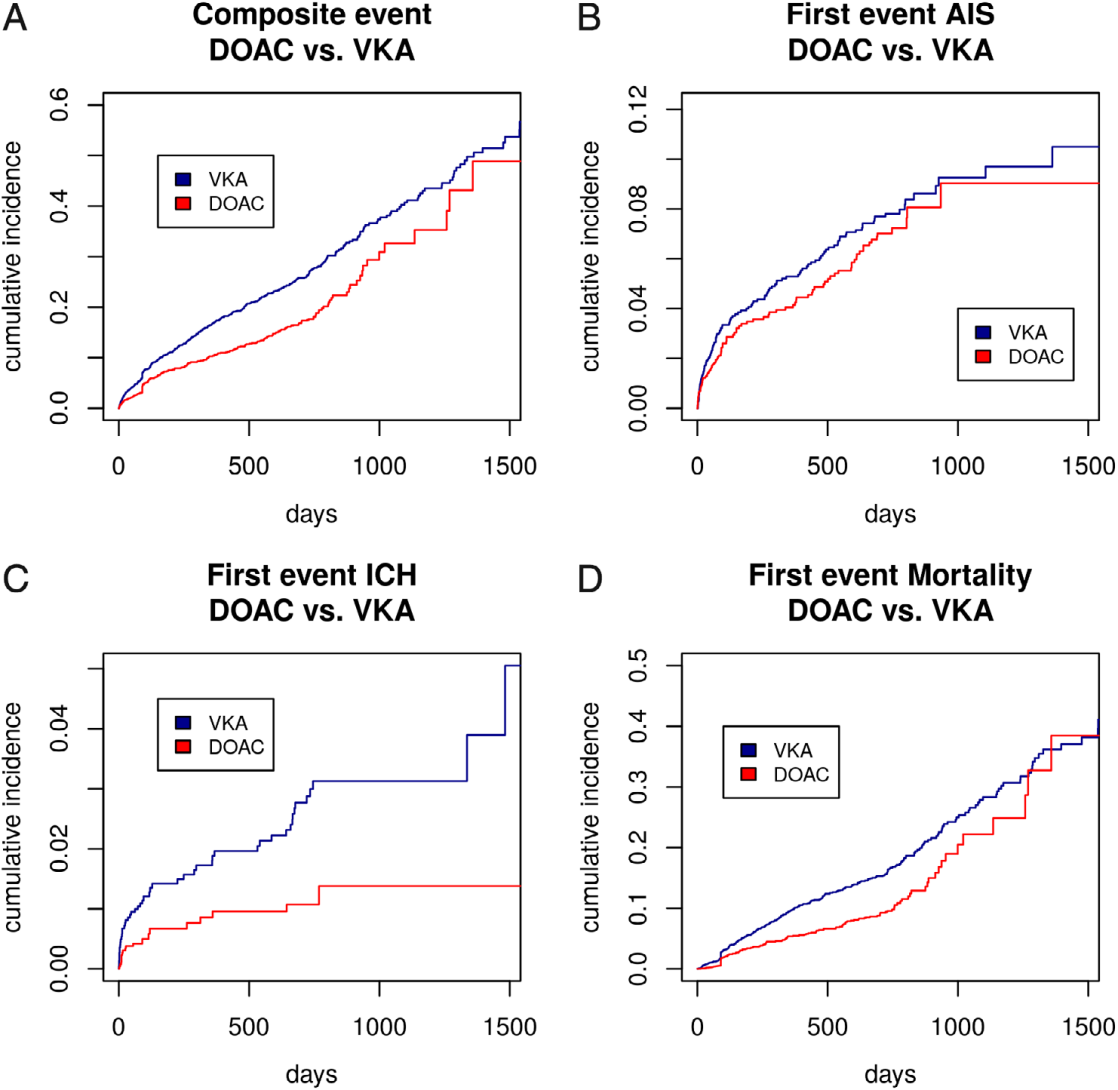
Kaplan‐Meier curves for the composite endpoint (A), recurrent AIS (B), ICH (C), and mortality (D) for patients in both groups (VKA vs DOAC). AIS = acute ischemic stroke; DOAC = direct oral anticoagulant; ICH = intracranial hemorrhage; VKA = vitamin K antagonist. [Color figure can be viewed at www.annalsofneurology.org]

Total follow‐up time was 5,970 patient‐years, 3,382 in the VKA group, and 2,588 in the DOAC group. During the entire follow‐up (see Fig 2), 262 patients had AIS (4.4%/year), 71 had ICH (1.2%/year), and 439 died (7.4%/year). Figure 2 displays the Kaplan‐Meier curves for the primary composite endpoint and its components. Overall, in 2,984 (61%) patients, the full data set of baseline characteristics was available and these patients were included in the main analysis. The following values were missing: history of ICH (1,467 missing values), NIHSS on admission (489 missing values), hypertension (22 missing values), diabetes mellitus (10 missing values), age (eight missing values), history of stroke or TIA (two missing values), and sex (one missing value).

DOAC treatment was associated with a lower risk of the composite primary outcome (HR, 0.82; 95% CI, 0.67–1.00; *p* = 0.05) compared with VKA (Table 3). Patients receiving DOAC were also at lower risk of ICH (HR, 0.42; 95% CI, 0.24–0.71; *p* < 0.01). Risk of recurrent AIS (HR, 0.91; 95% CI, 0.70–1.19; *p* = 0.5) and mortality (HR, 0.83; 95% CI, 0.68–1.03; *p* = 0.09; all Table 3) did not differ between DOAC and VKA treatment.

**Table 3:** Primary and Secondary Outcomes in Patients Taking DOAC Compared To Patients Taking VKA

	VKA n = 2,256	DOAC n = 2,656	HR (95% CI)	*p*
Primary outcome				
Composite endpoint^a^	485 events/3,207 patient‐years (15.1%/y)	272 events/2,479 patient‐years (11.0%/y)	0.82 (0.66–1.00)	0.05
Secondary outcomes				
Recurrent AIS	137 events/3,229 patient‐years (4.2%/y)	110 events/2,484 patient‐years (4.4%/y)	0.91 (0.70–1.19)	0.5
ICH	52 events/3,302 patient‐years (1.6%/y)	22 events/2,549 patient‐years (0.9%/y)	0.42 (0.24–0.71)	<0.01
Mortality	358 events/3,324 patient‐years (10.8%/y)	161 events/2,556 patient‐years (6.3%/y)	0.83 (0.68–1.03)	0.09

Given as number of events and total follow‐up time (annualized event rate).

a Composite endpoint: acute ischemic stroke (AIS), intracranial hemorrrhage (ICH), and mortality.

**Figure 3:**
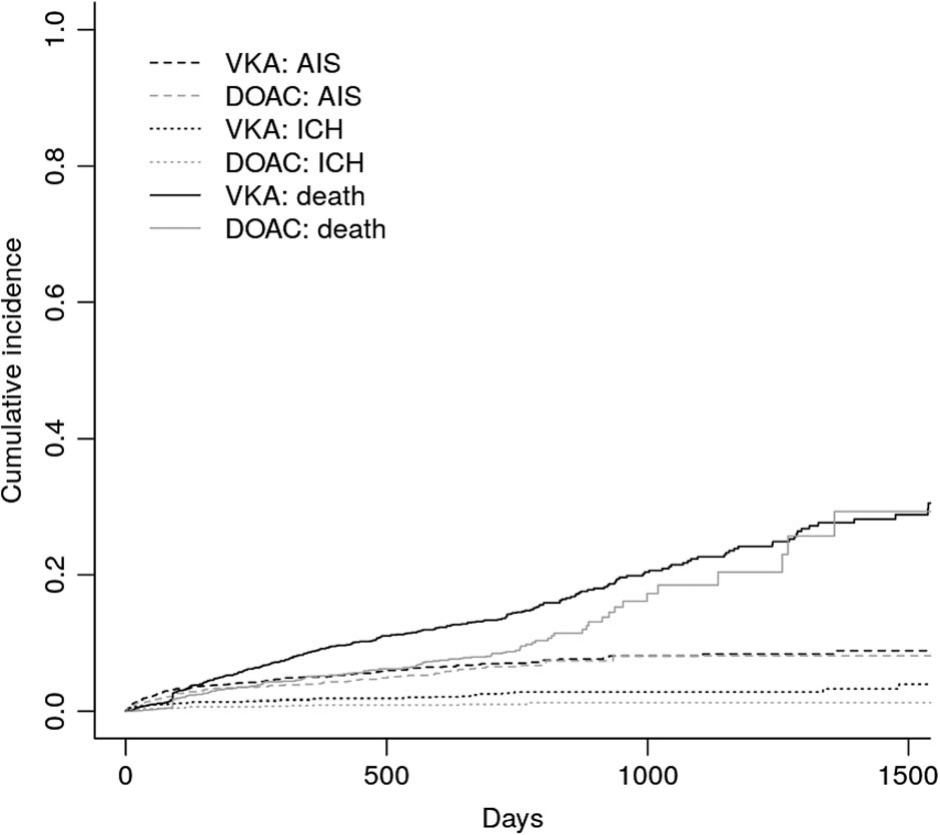
Cumulative incidence function. DOAC = direct oral anticoagulant; VKA = vitamin K antagonist.

The cumulative incidence function (Fig 3) does not suggest clear evidence for competing risks. In general, the highest hazard was the hazard for mortality. Patients treated with VKA were at higher risk for all events.

Using multiple imputation, results for the primary composite endpoint (HR, 0.85; 95% CI, 0.71–1.00; *p* = 0.05) and the secondary analysis of ICH (HR, 0.34; 95% CI, 0.19–0.62; *p* < 0.01) remained significant. No statistically significant difference was observed between patients with DOAC and VKA for risk of mortality (HR, 0.86; 95% CI, 0.70–1.06; *p* = 0.17) and the risk of recurrent AIS (HR, 0.89; 95% CI, 0.68–1.18; *p* = 0.42). PSM analysis found a significant difference in the primary composite endpoint (HR, 0.81; 95% CI, 0.70–0.93; *p* < 0.01), ICH (HR, 0.33; 95% CI, 0.19–0.58; *p* < 0.01), and mortality (HR, 0.72; 95% CI, 0.61–0.85; *p* < 0.01). Again, risk of recurrent AIS (HR, 1.09; 95% CI, 0.86–1.38; *p* = 0.46) did not differ between DOAC and VKA.

### *Post‐Hoc Analysis*

Among the 2,656 patients receiving a DOAC, 723 (27.2%) took apixaban, 959 (36.1%) dabigatran, 11 (0.4%) edoxaban, and 880 (33.1%) rivaroxaban, and in 83 patients (3.1%), type of DOAC was not specified. Compared to VKA, DOAC_bid_ (apixaban or dabigatran) was associated with a reduced risk of the composite endpoint (HR, 0.63; 95% CI, 0.48–0.82; *p* < 0.01). DOAC_od_ (edoxaban or rivaroxaban) had a lower hazard than VKA for the composite endpoint, but this was not statistically significant (HR, 0.83; 95% CI, 0.64–1.08; *p* = 0.16). Both DOAC_od_ and DOAC_bid_ were not associated with a reduced risk of recurrent AIS (DOAC_od_: HR, 1.39; 95% CI, 0.94–2.06; *p* = 0.10; DOAC_bid_: HR, 0.83; 95% CI, 0.55–1.28; *p* = 0.41). Both DOAC_od_ and DOAC_bid_ were significantly associated with a reduced risk of ICH (DOAC_od_: HR, 0.25; 95% CI, 0.07–0.84; *p* = 0.02; DOAC_bid_: HR, 0.42; 95% CI, 0.18–0.97; *p* = 0.04) and mortality (DOAC_od_: HR, 0.70; 95% CI, 0.50–0.97; *p* = 0.03; DOAC_bid_: HR, 0.52; 95% CI, 0.37–0.73: *p* < 0.01). Among patients with unknown type of DOAC (n = 83), mortality was high (41 death). For patients on VKA having recurrent AIS during follow‐up (n = 137), information on VKA at recurrent AIS was available in 65 patients (47.4%). INR was <2.0 in 45 of these 65 patients (69.2%) and ≥2.0 in 20 (30.8%). In patients on VKA having ICH during follow‐up (n = 52), INR at ICH was available in 26 patients (40.0%). INR was <2.0 in 13 patients (50.0%), 2.0 to 3.0 in 10 (38.5%), and >3.0 in 3 (11.5%).

### *Subgroup Analyses*

**Figure 4:**
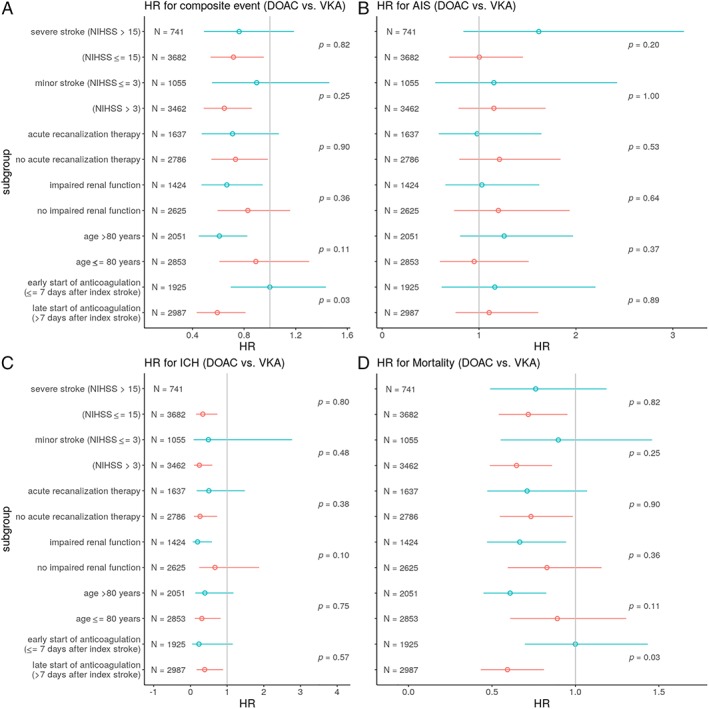
Subgroup analyses of treatment effect in predefined subgroups for the composite endpoint (A), recurrent AIS (B), ICH (C), and mortality (D). AIS = acute ischemic stroke; DOAC = direct oral anticoagulant; HR = hazard ratio; ICH = intracranial hemorrhage; NIHSS = National Institute of Health Stroke Severity Scale; VKA = vitamin K antagonist. [Color figure can be viewed at www.annalsofneurology.org]

Results for the predefined subgroups are displayed in Figure 4. There were significant modifications of treatment effect on the composite endpoint and mortality in patients with impaired renal function in favor of DOACs. There were no significant interactions between overall treatment effects in the other predefined subgroups.

## Discussion

This individual patient data analysis of seven international, independent cohort studies including 4,912 patients treated with oral anticoagulation after recent cerebral ischemia related to AF revealed the following major findings: First, treatment with DOACs commenced a median of 5 days after the index event has a lower risk of adverse outcomes compared to treatment with VKA; second, this benefit is mainly attributed to lower risks of ICH; and, third, the benefit is consistent across subgroups, including: those with either minor (NIHSS ≤3) or severe (NIHSS >15) strokes at baseline; those aged 80 years or older; those started on anticoagulants ≤7 days since index stroke; and those treated with acute recanalization therapies for the index stroke.

Our pooled data provide the largest available data set of best‐practice stroke unit patients with AF and recent cerebral ischemia treated with DOAC or VKA for secondary prevention. Subgroup analyses from the large RCTs comparing DOACs and VKAs showed consistent results in patients with a history of ischemic stroke compared to the overall trial population,^14^, ^15^, ^16^, ^17^ but patients with an ischemic stroke were excluded from these RCTs for at least 7 to 14 days and up to 3 to 6 months because of concerns of hemorrhagic transformation.^11^ In ARISTOTLE^7^ (comparing apixaban with VKA), 19% of all patients had history of ischemic stroke and only 33% were included within 1 year after index stroke.^15^ In ROCKET‐AF^8^ (comparing rivaroxaban with VKA), where 55% of all patients had a history of previous ischemic stroke, the median time from stroke to enrollment in the trial was 551 days.^16^ To the best of our knowledge, no information on the delay between ischemic stroke and trial enrollment is available for RE‐LY (Randomized Evaluation of Long‐term Anticoagulation Therapy)^5^, ^14^ (comparing dabigatran with VKA, 20% of patients had a history of ischemic stroke) or ENGAGE‐TIMI AF 48 (Effective Anticoagulation With Factor Xa Next Generation in Atrial Fibrillation–Thrombolysis in Myocardial Infarction 48)^6^, ^17^ (comparing edoxaban with VKA, 28% of patients had a history of ischemic stroke). By contrast with RCTs, all patients in our cohort had a recent ischemic stroke within the last 3 months preceding the start of anticoagulation and 81% within 14 days. The median delay of just 5 days in both—patients receiving DOAC and those receiving VKA—means that 80% of our patients would not have been eligible for any of the pivotal RCTs.^43^

We can only speculate on the reasons for the apparent benefit of DOAC therapy in patients with a recent ischemic stroke and AF, but it might be related to the increased risk of ICH and mortality,^9^, ^10^ making them ideal candidates for DOAC therapy. A post‐hoc analysis found that the reduced risk of ICH—and mortality—was consistent across different types of DOACs (od and bid intake). For the risk of AIS, neither DOAC_bid_ nor DOAC_od_ were significantly superior to VKA. However, only DOAC_bid_, but not DOAC_od_, were associated with a significantly reduced risk of the composite endpoint compared to VKA. We urge caution not to overinterpret these finding. Despite multivariate analysis, residual confounding is likely to account for the differences in these nonrandomized comparisons. In patients on VKA, 70% of the recurrent AISs occurred in patients with INR <2.0 whereas only 10% of the ICH occurred in patients with INR >3.0. A recent study found that in patients on rivaroxaban with ICH, rivaroxaban plasma levels were high in only 25% of the patients.^44^ These findings underline that other causes of ICH beyond anticoagulant activity (ie, small vessel disease) might play an important role in ICH associated with the use of oral anticoagulants.

Our interaction analysis showed that the timing of initiation of oral anticoagulation (≤7 versus >7 days) did not significantly modify the association between the type of oral anticoagulation (DOAC versus VKA) and the endpoints. The lower risk of ICH under DOAC compared to VKA treatment was therefore not modified by the timing of initiation of anticoagulation. This aligns with our previous findings showing that an early start of DOAC (≤7 days from index stroke) is associated with a low risk of ICH.^23^ However, the timing of initiation of oral anticoagulation still remains unclear and is being investigated in ongoing controlled clinical trials.

Our new analyses expand on the previous observations by exploring the effects of DOACs compared to VKAs across different subgroups. We have shown that the overall benefit of DOACs in patients with a recent ischemic stroke are consistent in patients with both minor and major stroke, patients treated with acute recanalization therapies for the index event, and in older patients. These findings are reassuring, because patients with major strokes and of older age might have an increased a priori risk of ICH and mortality that could be aggravated by anticoagulation. Interestingly, we found evidence of a potential additional benefit of DOAC therapy in patients with impaired renal function. In this subgroup, use of low‐dose DOACs might have contributed to reduced risks of ICH and mortality, but this remains speculative. Whether the potential beneficial effect of DOACs in patients with impaired renal function is explained by the over‐representation of patients with severely impaired renal function (<30ml/min) in the VKA group (given that this is a contraindication for DOAC therapy) needs to be further studied. We therefore emphasize caution in the interpretation of our results.

Our study has the following strengths: (1) We conducted an individual patient data analysis of seven international studies involving patients from Europe and Asia, which makes our results broadly generalizable; (2) we report on a large data set of patients with a recent ischemic stroke currently available with nearly 5,000 patients and a total follow‐up time of 5,970 patient‐years; (3) all participating studies prospectively recruited stroke patients (in the majority of cases consecutively) minimizing selection bias; (4) both cohorts—VKA and DOAC patients—had a comparable size of 2,500 patients each, a comparable delay between index stroke and start of anticoagulation of only 5 days, and were well balanced in most of the known risk factors and demographics; and (5) sensitivity analysis using PSM and cumulative incidence function did not identify competing risks and using multiple imputation for missing variables confirmed the main findings of our study—a reduced risk of the composite endpoint as well as a reduced risk of ICH. The reduced risk of ICH and mortality compared to VKA was consistent among all types of DOACs (od and bid).

Nevertheless, our study has some limitations: (1) We report on an observational, nonrandomized study rather than a randomized trial. Allocation to the type of oral anticoagulant is likely to be affected by biases that cannot be fully adjusted for, and the results have to be interpreted with great caution; RCTs need to provide further data on the risks and benefits of early start of DOACs in patients with AF. (2) Reasons for the choice of the type of anticoagulation and, in case of DOAC treatment, for the use of a specific agent were not recorded; it is likely that these choices were influenced by unmeasurable factors related to the individual physician's decision, which might have influenced our key findings. (3) Although most risk factors and baseline characteristics were well balanced between patients receiving VKA and DOAC, there were significant differences between both cohorts with patients on DOACs having a lower baseline NIHSS, a higher treatment rate of intravenous thrombolysis, and lower prevalence of diabetes. Although we adjusted for these characteristics, residual confounding by patient characteristics that were not measured in these cohorts cannot be ruled out (ie, patients’ choice, compliance and adherence with treatment). (4) Extracranial (ie, gastrointestinal) bleeding events have been found to be more frequent in patients taking DOACs.^2^ Collection of extracranial bleeding events and cause of death were heterogenous among the participating studies, and we therefore refrained from analyzing this data. (5) The majority of patients on DOACs received dabigatran, rivaroxaban, or apixaban. Only a minority of patients were treated with edoxaban, which limits the validity of our results for edoxaban. (6) In a minority of patients (3.1%), the type of DOAC was unknown, and this subgroup had a higher mortality than the rest of the cohort. (7) We did not collect information on time in therapeutic range for patients on VKA. Patients on VKA having recurrent AIS had subtherapeutic (<2.0) INR values in 70% of the cases, indicating that poor compliance is a potential factor. However, INR was supratherapeutic (>3.0) in only 10% of the patients having ICH. (8) Finally, we did not account for therapy changes during follow‐up that might have influenced our results.

Currently, several RCTs are investigating the risks and benefits of early start of DOACs^13^ (Switzerland: ELAN ClinicalTrials.gov Identifier: NCT03148457; Sweden: TIMING ClinicalTrials.gov Identifier: NCT02961348; UK: OPTIMAS: EudraCT, 2018‐003859‐38 and USA: START ClinicalTrials.gov Identifier: NCT03021928). Our findings support these trials, which do not compare DOACs with VKAs, but will give further insights into what is the optimal time point to start DOACs after a recent stroke. The results of our study underline the importance of RCTs further investigating early timing of DOACs in patients with AF and a recent ischemic stroke.

To summarize, our study provides class IIa and level B evidence that DOAC therapy commenced early after a recent ischemic stroke in patients with AF is beneficial compared to VKA therapy. This benefit is driven by a decreased risk of ICH.

## Author Contributions

D.J.S., S.T.E., and G.M.D.M. contributed to the conception and design of the study. All authors contributed to the acquisition and analysis of the data. D.J.S., S.S., S.T.E., and G.M.D.M. contributed to drafting the text and preparing the figures.

## Potential Conflicts of Interest

The following companies manufacture drugs involved in this study: Bayer (BY, rivaroxaban), Boehringer Ingelheim (BI, dabigatran), Pfizer/Bristol Meyer Squibb (PB, apixaban), and Daiichi Sankyo (DS, edoxaban). D.J.S.: scientific advisory boards: BY and PB. M.P.: honoraria as a member of the speaker bureau of BI, BY, and PB. M.K.: speaker honoraria from DS, BY, and PB. K.M. speaker honoraria from BY, travel grant from PB. L.H.B.: consultancy or advisory board fees or speaker's honoraria from BY, PB. K.W.M.: participated in advisory boards for DS and BY; member of trial steering committee for BY‐sponsored NAVIGATE‐ESUS trial. G.T.: scientific advisory boards: BY, DS, and BI. S.A.: speaker honoraria from DS, BI, and BMS/PB. H.Y.: speaker honoraria from DS, BY, BI, and BMS/PB. Research funding: PB. S.S.: employment by BY HealthCare Pharmaceuticals’ Japanese subsidiary, BY Yakuhin, from January 2018 (all his contributions to this study were made during his employment at the National Cerebral and Cardiovascular Center). N.P.: advisory boards BI, BY, and DS. K.T.: speaker honoraria from DS, BY, BI, and PB. P.A.L.: scientific advisory boards: BY, DS, and BI. Funding for travel or speaker honoraria: BY and BI. Research funding: BI. D.J.W.: speaking honoraria: BY. S.T.E.: funding for travel or speaker honoraria: BY and BI. Scientific advisory boards: BY, BI, and PB. Educational grant from PB. G.M.D.M.: travel honoraria: BY; speaker honoraria: PB.

## Supporting information


**Supplemental Tables**
